# Strategies for improving treatment retention for buprenorphine/naloxone for opioid use disorder: a qualitative study of issues and recommendations from prescribers

**DOI:** 10.1186/s13722-024-00516-z

**Published:** 2024-11-21

**Authors:** Gary J. Young, Leonard D. Young, Md. Noor-E-Alam

**Affiliations:** 1 Center for Health Policy and Healthcare Research, Northeastern University, Boston, MA USA; 2 Bouve College of Health Sciences, Northeastern University, Boston, MA USA; 3 D’Amore-McKim School of Business, Northeastern University, Boston, MA USA; 4 Prescription Monitoring Program, Massachusetts Department of Public Health, Boston, MA USA; 5 Department of Mechanical and Industrial Engineering, College of Engineering, Northeastern University, 360 Huntington Avenue, Boston, MA 02115 USA

**Keywords:** Opioid use disorder, Buprenorphine, Qualitative research, Substance use disorder, Chronic disease management

## Abstract

**Background:**

Opioid use disorder (OUD) remains a significant public health issue as the number of opioid-related overdose deaths continues to reach new highs each year. Buprenorphine/Naloxone is a medication that has been shown to be highly effective for the treatment of OUD. However, the clinical management of patients on this medication is challenging as many patients discontinue treatment prematurely. We conducted a qualitative study focusing on experienced prescribers of buprenorphine to learn about what they believe are key challenges in prescribing this medication to patients with OUD and related strategies for improving treatment outcomes.

**Methods:**

We conducted two rounds of interviews with 12 prescribers who were either trained as a primary care physician, nurse practitioner, or physician assistant. These prescribers were recruited from an academically-based treatment program, a community health center, and a commercial substance use disorder treatment facility. Interview data were coded and analyzed in accordance with a grounded theory approach.

**Results:**

Key findings and related recommendations emerged for patient monitoring, integration of behavioral health with prescribing, patient volume requirements, and use of telehealth.

**Conclusion:**

The interviews generated a number of recommendations for improving patient outcomes from buprenorphine treatment. Some of these recommendations can be implemented quite readily whereas others entail more substantial resources and time commitments.

## Background

Opioid use disorder (OUD) continues to be a significant public health challenge directly impacting several million individuals in the US [1–6]. COVID-19 worsened the situation by contributing to a substantial rise in overdose-related deaths as individuals who were struggling with addiction became more isolated and faced significant barriers to obtaining treatment and social services [7] Buprenorphine/Naloxone with the brand name Suboxone^®^ (hereinafter buprenorphine) is one of three medications approved by the U.S. Food and Drug Administration (FDA) for OUD [8–12]. Buprenorphine has been shown to lessen the withdrawal symptoms associated with opioid addiction and to reduce the likelihood that an individual will experience a fatal overdose [13, 14]. Over 1.5 million individuals in the US were prescribed this medication in 2021 [15].

At the same time, treatment retention for buprenorphine has been a serious problem. Studies indicate that more than 50% of patients who begin this medication discontinue well within 12 months, which exposes them to significant risk for returning to use, hospitalization, and overdose [16–19]. Several studies have used administrative data to identify patient- and prescriber-level characteristics that are associated with treatment discontinuation [16–21]. In particular, the research shows that patients who are younger in age, present with chronic physical and mental health conditions, and have a history of non-opioid substance use disorder are more likely to discontinue treatment prematurely. Studies also indicate that patients who begin medication treatment at relatively lower doses of buprenorphine are more likely to discontinue. Qualitative research has also been undertaken in the form of patient interviews to obtain a more in-depth understanding of the factors that can promote or impede treatment retention [22–24]. These studies indicate that treatment retention is associated with a patient’s readiness to commit to treatment, comfort with a prescriber’s interpersonal style, and perception of being well supported by treatment staff for logistical matters such as scheduling refill appointments.

Considerably less research has focused on the role of providers in patients’ treatment retention. One of the few studies to address this topic used a mixed-methods approach to explore provider-level factors in relation to treatment retention for patients [21]. The results of this study suggest that a provider’s clinical training and practice setting are associated with patients’ treatment retention. More specifically, study results suggest that providers who impose fewer treatment restrictions on patients (e.g., required counseling, negative drug tests) achieve better retention rates. These findings highlight the important role of providers in the treatment retention of patients with OUD. At the same time, there is limited research seeking providers’ perspectives on why patients discontinue buprenorphine treatment and what can be done to improve retention. In this manuscript, we report results from a qualitative research study for which we sought prescribers’ views on challenges and opportunities for improving buprenorphine treatment retention for patients with OUD.

## Methods

This was a qualitative study focusing on clinicians who were experienced prescribers of buprenorphine for individuals with opioid use disorder. Data collection entailed two components: (1) an initial round of individual interviews with prescribers to identify common problems/issues for managing patients undergoing buprenorphine treatment, and (2) a subsequent set of focus groups with the participating prescribers to identify opportunities for addressing key treatment issues and concerns that were identified during the first round of interviews. For conducting the study, we followed closely the recommended guidelines of the Standards for Reporting Qualitative Research [25].

### Study sample

We assembled a group of prescribers representing different types of clinical professionals and settings. Through contacts at several clinical facilities in Massachusetts that treat patients with OUD, we reached out to 17 prescribers inquiring about their interest in participating in the study. The clinical sites comprised an academic medical center, an urban safety net hospital, a community-based hospital, and an independent substance abuse treatment organization. Twelve prescribers agreed to participate with two or more represented from each clinical site. Among the twelve, 7 were medical doctors, 3 were nurse practitioners, and 2 were physician assistants. Table 1 presents aggregate demographic and practice-related information for the participating prescribers (years of experience, panel size etc.).

**Table 1:** Demographic and clinical profile of study

Participants (*n* = 12)	
**Demographic Characteristics**	
Prescribing Experience (mean — years)	17.4
Gender (# male)	8
**Professional Training**	
M.D.	7
N.P.	3
P.A.	2
**Clinical Setting**	
Academic Medical Center	3
Urban Safety Net Hospital	4
Community Hospital-based Program	2
Independent Substance Abuse Program	3

### Data collection

As noted, we conducted two rounds of data collection with participating prescribers; a round of individual interviews and a subsequent round of focus groups. In the first round we conducted a 60-minute interview with each prescriber to obtain information about their training and education, experience prescribing buprenorphine, and views about the challenges of managing patients on buprenorphine to retain them in treatment to achieve a positive outcome. Two members of the research team conducted each individual interview by following a semi-structured questionnaire format. We also used round one to assess whether the 12 prescribers would constitute a sufficient sample in terms of capturing a range of themes for round two or whether we would need to recruit additional prescribers for the study.

The second round comprised four focus groups that were organized around themes that emerged during the first round of interviews regarding challenges/issues for managing patients undergoing buprenorphine treatment. Each focus group comprised three or more prescribers and was between 90 and 120 min in duration. We randomly assigned prescribers to one of the four focus groups.

The focus groups were not intended to necessarily achieve consensus among prescribers on issues raised during the individual interviews, but rather to expose the prescribers to the different perspectives that had been raised by other participating prescribers during the first round of interviews and generate recommendations to improve treatment retention. The same member of the research team moderated each focus group. This study was approved by a Northeastern University Institutional Review Board (IRB 19-05-06). All prescribers provided informed consent for participation in this study.

### Analysis

The audio recordings for the individual interviews and the focus groups were transcribed. NVivo 10 software was used to assist with data coding, management, and analysis. For the interviews, in accordance with a grounded theory approach, members of the research team read, analyzed, and coded the transcripts [26–28]. The coding and analysis were conducted iteratively. Specifically, we applied open coding to the transcripts during the interview process. This step entailed iterative reviews of the transcripts, development of preliminary code lists, and coding of the data to identify emerging patterns relevant to study objectives in line with the constant comparison method [27]. Following completion of the interviews, a final set of open codes was produced that were applied to all transcripts. As a subsequent step, we generated a set of axial codes to identify key themes from the interviews.

These steps were repeated for analyzing the transcripts from the focus groups for purposes of identifying strategies and recommendations. The proposed recommendations of the focus groups were compared and contrasted along with the quotes that most accurately illustrated the themes. To assess interrater reliability, two members of the research team independently coded a sample of 5 interview transcripts using the final set of codes and results were compared. The percent agreement was 86% and Kappa was 72%.

## Results

Four main themes emerged from the individual interviews that were further explored and discussed during the focus groups: (1) availability of systems and tools for managing patients, (2) integration of behavioral health with prescribing, (3) implications of patient volume for effective patient management, and (4) role of telehealth for refill/medication appointments. The interviews from round one revealed substantial consensus among the prescribers as to these themes such that we were confident that we had achieved sufficient saturation in data collection and no additional prescribers were needed for the study sample. The results are summarized in Table 2.

**Table 2:** Summary of findings

Theme	Issues	Consensus recommendations
Systems and tools to support patient management.	Lack of tools and staff support for tracking patients. Inadequate tools for assessing patient fit with medication treatment options.	States adopt alert system as part of prescriber monitoring system for missed prescription refills. Increase investment in developing decision-support tools for assessing the best treatment options for a patient.
Integration of behavioral health with prescribing.	Whether counseling should be required. Inadequate communication between prescriber and counselor. Timely access to psychiatrists.	Professional associations establish a policy that counseling should be encouraged but not required. Treatment programs proactively secure patients’ permission for information exchange between prescriber and counselor. States establish a web-based clearinghouse for psychiatry services.
Implications of patient volume for effective patient management.	Whether volume thresholds are useful.	State agencies offer guidance to prescribers on appropriate patient volume based on resource availability.
Role of telehealth.	Whether telehealth can fully replace in-person visits for prescriptions.	Professional associations recommend continuation of periodic in-person visits as part of the prescribing schedule.

### Systems and tools for managing patients

During the individual interviews a central theme centered on the availability of systems and tools for managing patients undergoing buprenorphine treatment. Many of the prescribers reportedly worked in settings where there was little infrastructure to support patient management for those undergoing buprenorphine treatment. They lacked patient registries, they lacked reliable systems for patient communication, and they lacked alerts for missed medication refill pickups. In fact, more than half the prescribers reported that they worked largely alone with essentially no support staff and no digital resources for managing patients with OUD. Even prescribers who did work in settings where staff support and other resources were available reportedly lacked the information to reach out in a timely way to patients who were discontinuing treatment prematurely.

Prescribers also spoke about challenges for helping patients decide among medication options for treating OUD, specifically buprenorphine versus methadone. Prescribers reported that they guided patients based on their clinical judgement focusing on such considerations as a patient’s previous experience with a medication. While prescribers expressed confidence that they could effectively guide most patients in their decision making, some indicated a desire for well- developed decision tools that could be used for assessing which medication option for a given patient is more likely to lead to a successful outcome.

During the focus groups, several points of discussion and recommendations were advanced. One was the view that many prescribers would likely benefit from systems that alerted them to missed refill appointments for their patients. It was suggested that state administered prescription monitoring programs (PMPs), such as the Massachusetts Prescription Awareness Tool (MassPAT), be equipped to alert prescribers to missed medication appointments. Prescribers suggested that they receive alerts if one of their patients fails to pick up medication. As an additional consideration, several of the prescribers noted such an alert would be most helpful if it could also be directed to their support personnel (i.e., delegates) rather than themselves given all the other alerts and prompts they deal with in their daily practice.

When my patients begin to miss one or two refills, it is usually a bad sign that they’re in trouble. But I often do not become aware that they’re not picking up their meds until it is probably too late. They will also likely not be showing up for their refill appointments, but I have a lot of patients and they just slip through unnoticed. An alert from the PMP would certainly help.

Another recommendation was for more development of decision tools for assessing patient fit with medication options. Prescribers acknowledged that some tools do exist, but were of the opinion that they lack the breadth and depth of information that they themselves believe is relevant to assessing whether a patient is best suited for buprenorphine vs. methadone. For example, prescribers believed information on socio-economic status, family support, and travel requirements are all important to consider along with a patient’s clinical profile. Prescribers also noted that better tools would likely entail routine collection of more information about patients prior to treatment decisions than is currently the case.

Certainly there are tools out there, you know for predicting [opioid] dependence and selecting medication options…but they are still pretty primitive from a clinical standpoint. They offer some guidance but you need to rely heavily on your own experience and clinical intuition, which is fine and appropriate. But sure if better tools come along this will be helpful assuming the necessary information can be obtained from patients for implementing them.

We are still in need for better systems to manage patients with addiction, OUD in particular. When you consider how often these patients fall out of treatment with Suboxone, we need to think about who will benefit from this medication and who is better situated for methadone where treatment is provided in a more structured setting.

### Integration of behavioral health with prescribing

Behavioral health emerged as a theme during the individual interviews with respect to its value for improving treatment retention. In this vein, several issues arose regarding counseling specifically. One was whether counseling should be required as part of buprenorphine treatment. Some of the prescribers worked in clinical settings where counseling is required as a component of the treatment program whereas others worked in settings where counseling was strictly voluntary though encouraged.

Another issue was communication between prescribers and counselors. The majority of prescribers worked in settings where counseling services were not integrated with the prescribing program. In such settings, communication between prescribers and counseling was reportedly problematic. Before prescribers could communicate with a patient’s counselor or access notes from counseling sessions, the patients needed to sign a waiver in compliance with HIPPA privacy requirements. Prescribers commented that waivers were not routinely obtained and thus they faced a barrier to obtaining potentially valuable information about their patients that might have helped them manage their buprenorphine treatment. Further, some prescribers felt strongly that counselors and prescribers should engage in regular two-way communication about patients so that counseling sessions can be used to address specific emotional barriers to treatment retention. Indeed, there was a general impression among prescribers that counseling for OUD often followed a somewhat generic format that did not sufficiently target issues that were most relevant for their patients who were struggling to remain in treatment.

It really is important that we communicate with counselors when our patients are in treatment. It is a very vulnerable time for them and if they are in counseling, they need their prescriber and counselor to be fully informed about all aspects, emotional and physical, of their treatment status.

This really has been a point of frustration for me that I often cannot obtain information from my patients’ counselors. Sometimes HIPPA rules are the issue and other times maybe nothing but poor coordination across systems. But, either way it is an impediment to the best care I can provide to these patients.

One additional issue concerned timely access to psychiatrists. Prescribers reported that they often had difficulty securing such services for treating patients who had comorbid conditions, including bipolar and major depression, that impeded treatment retention and likely required additional psychopharmacology that they lacked the training to manage.

Referral for psychiatry is an issue because the wait times are really long right now, the longest I have seen in the more than 20 years I have been practicing. For these patients [those with OUD] they are very vulnerable and their mental health conditions are a significant complicating factor to medication treatment.

The focus groups generated several recommendations regarding counseling and its connection to managing patients on buprenorphine. One was that counseling for individuals undergoing buprenorphine therapy should be encouraged but not required. This recommendation was firmly endorsed even among prescribers who worked at clinical sites where counseling was required. While all prescribers saw value in counseling for supporting patients during buprenorphine treatment, they did not believe it should be a condition of buprenorphine treatment. Prescribers agreed that no definitive evidence currently exists that counseling is strongly associated with treatment retention and outcomes. Most importantly, they believed that it was important to refrain from creating any additional barriers for patients to obtain medication therapy. They were of the opinion that such a requirement deters some patients from starting buprenorphine treatment. As one participant noted:

I cannot count how many times I have had a patient decline treatment [buprenorphine treatment] because they did not want to enter into counseling. Some say they do not have the time, others feel they will be judged or are embarrassed to discuss their situation. But whatever the reason, they decline treatment and this is a very poor result.

Another recommendation was for treatment programs to offer counseling services that are patient centered in both format and schedule. Prescribers recommended a more systematic approach to securing waivers for HIPPA to facilitate the exchange of information between prescribers and counselors that can foster patient centered care. It was suggested that waivers, whether administered in paper form or digitally, be presented to patients at the time of an initial medication appointment for buprenorphine. In addition, prescribers recommended that treatment protocols include a provision calling for prescribers and counselors to be able to exchange information about patients undergoing treatment for OUD.

One additional recommendation related to the availability of psychiatry services for patients with OUD emerged. Prescribers recommended that states establish a web-based clearinghouse for psychiatry services for supporting patients with OUD. This website would provide the names, availability, and contact information of psychiatrists who were willing to provide consultation services for OUD patients within a short time frame.

### Treatment implications from patient volume

Patient volume emerged as a theme in terms of what, if any, volume limits were needed for prescribers. During individual interviews, some prescribers raised the question about whether a high volume of OUD patients impeded a prescriber’s ability to effectively manage patients struggling to remain in treatment. At the same time, most of the prescribers felt they could effectively manage more patients than federal policy had permitted before the recent elimination of volume requirements.

During the focus groups, prescribers agreed that uniform volume limits were not appropriate given the need to expand access to buprenorphine treatment for patients with OUD. Most prescribers held the view that prescribing buprenorphine from a purely pharmacological perspective was no more difficult than prescribing other medications. The challenging aspect of prescribing buprenorphine was managing patients so they adhered to their medication schedule and avoided illicit drugs.

As such, prescribers believed the appropriate patient volume depends on the support systems that a prescriber has in place.

I do not see much difference in prescribing buprenorphine than I do medications for most other conditions. But certainly dealing with the cravings my patients experience and the fact that many of them are living on the fringes regarding employment and family, that makes my job really hard. Certainly, a high volume of these patients presents challenges, but it really depends on what you have for support systems.

Patients receiving [buprenorphine] treatment are always struggling with their addiction and while the medication is valuable clinically, it is often not enough. They need a great deal of support and that entails outreach, sometimes behavioral health, and transportation support if available. You also need to be able to monitor their refill schedule and medication adherence. One person cannot do all of this; you need a dedicated team. But no question many prescribers are pretty much on their own.

Prescribers recommended that state agencies should consider assuming responsibility for providing guidance to prescribers regarding appropriate volume limits and related matters for managing patients. In particular, prescribers suggested that states assemble information for prescribers to consider in their decisions about patient volume. This initiative might also include establishing certification programs that are available to prescribers that feature elements of evidence-based practice for managing patients and suggested volume levels relative to practice setting and resources.

### Role of telehealth

The opportunities and challenges associated with using telehealth technology for prescribing buprenorphine and related refill appointments came up prominently during the individual interviews. Most of the prescribers relied on telehealth for appointments during the pandemic. More than half reported that they planned to continue offering this technology to patients as a means of promoting access to medication treatment for this patient population.

Still, most prescribers had reservations about offering patients refill appointments entirely through telehealth technology. During the individual interviews, most prescribers characterized telehealth as a mixed bag for purposes of conducting buprenorphine refill appointments. While prescribers believed that telehealth expanded patients’ access to refill appointments, there was much agreement that virtual appointments were not as effective as in-person meetings for assessing a patient’s status in terms of how they are tolerating treatment. Telehealth was reportedly not helpful for reading body language and other visual cues, which were noted as valuable indicators of patients’ status.

Patients starting treatment [with buprenorphine] are often still heavily in the throes of addiction and this makes them very vulnerable for discontinuing treatment and relapse. I can judge a great deal on how they are coping by talking to them and seeing them in person, their affect and body language. Much less so during a telehealth appointment. In-person appointments remain important even with better remote technology.

As I saw it telehealth for prescribing buprenorphine was an emergency response. I understand that now we have this [virtual technology] in place for these appointments, we may not want to return entirely to in-person visits but I can tell you that telehealth is not a full substitute. I am less able to assess patient progress this way and also coordinating their tests [urinalysis] is difficult.

Also, prescribers noted that when patients are receiving their refills through telehealth appointments, it is more difficult to coordinate their testing for the presence of illicit opioids. Most prescribers reported that when relying on telehealth during the pandemic their patients did not undergo routine testing.

While the focus groups did not produce consensus among prescribers as to the degree to which telehealth could reliably replace in-person meetings, prescribers did agree that refills should not be entirely conducted through telehealth visits. They recommended a hybrid model whereby possibly 1 in every 4 refill appointments would be in person or refill appointments would be conducted in person until patients have achieved a period of stability in their treatment. Also, prescribers noted that an increasing reliance on telehealth enhances the importance of having other systems in place for monitoring patients’ progress.

A balance needs to be struck between in-person and virtual but our recommendation should be to lean toward in person. I recognize that telehealth promotes access and convenience but these patients have certain clinical issues that warrant they be monitored closely and often in person.

## Discussion

Opioid use disorder is a significant public health problem that continues to impact the lives of millions of individuals, both physically and emotionally. While buprenorphine is a key medication for treating opioid use disorder, a major challenge is retaining individuals in treatment.

Our qualitative study of experienced prescribers highlighted a number of issues that potentially affect treatment retention for patients with OUD. During the initial interviews, prescribers raised important questions and opinions on a wide number of topics that largely centered around several themes pertaining to systems and tools for patient management, behavioral health and treatment retention, patient volume requirements, and the role of telehealth for prescribing buprenorphine. While prescribers did differ on certain issues, differences could often be reconciled, and the focus groups proved useful for identifying strategies and recommendations that could be endorsed by most if not all prescribers.

Some of the recommendations and strategies seem to be easy to implement within a short timeframe, others less so. Those that should be readily implementable include wide-scale adoption by treatment programs of patient registries, systematic adoption of HIPPA waivers and related procedures to promote communication between prescribers and counselors, and dissemination by states and professional associations of evidence-based practices for managing patients undergoing buprenorphine treatment. Recommendations for states to establish prescriber certification programs, adoption of alerts for missed medication pick-ups, and development of web-based clearinghouses for psychiatry services are more likely to be longer term undertakings. Also, as of yet no permanent policy for prescribing buprenorphine via telehealth appointments exists as this is a contentious matter within the treatment community [29].

Some of the proposed recommendations might be seen to be in conflict with one another in terms of their impact on treatment barriers. In particular, while prescribers were uniformly opposed to mandatory counseling as they saw this as a treatment barrier, they also had reservations about prescribing buprenorphine primarily through telehealth appointments to promote treatment access due to concerns about whether a patient’s treatment progress can be properly assessed remotely. Although the evidence on telehealth and buprenorphine treatment for OUD is limited at this time, there is growing research suggesting that this mode of delivering buprenorphine is associated with a high degree of patient satisfaction as well as increased treatment retention [30, 31]. Still, the stated concerns of prescribers about telehealth points to possible future challenges in balancing treatment access with treatment effectiveness as certain treatment policies that potentially enhance access may also present risks for long-term treatment effectiveness. Certainly, research will be needed in the future to address such matters.

However, an underlying consideration for all the strategies and recommendations that prescribers advanced is that as a society we do not approach OUD in the same way we do other chronic health conditions. This was noted by many of the prescribers during the individual interviews and focus groups. Patient registries, alerts (reminders), and related decision tools have become widely adopted for managing conditions such as diabetes and congestive heart failure [32]; by comparison the pace of adoption for these clinical tools for OUD treatment seems strikingly slow. An added consideration is the fact the many of the prescribers participating in the study, including from a high profile academic medical center, appear to carry out treatment programs for individuals with OUD with little or no support in terms of personnel and technology.

Our study has two limitations that should be noted. First, while our study comprised a sample of clinicians with substantial prescribing experience, the findings reported are nonetheless drawn from a convenience sample that we assembled from outpatient treatment clinics in Massachusetts. As such, these clinicians’ attitudes toward buprenorphine treatment for OUD may not be representative of clinicians elsewhere who are engaged in medication treatment for this patient population. Second, some members of the study sample may have been reluctant to be fully candid about their views regarding buprenorphine treatment during the focus groups due to concerns about how they might be perceived by the other participants in the same focus group.

## Conclusions

In conclusion, we hope to see more research and policy initiatives address the challenging issue of poor treatment retention for patients receiving buprenorphine and other medications for OUD. Since this country began to confront the opioid epidemic, important advances have been made in the availability of medications for OUD treatment. However, these advances have limited value unless patients can remain in treatment to achieve a positive outcome.

## Data Availability

Not applicable.

## Abbreviations

OUD: Opioid use disorder
FDA: Food & Drug Administrations
PMP: Prescription monitoring program
MassPAT: Massachusetts Prescription Awareness Tool
